# Ultrasensitive detection of aggregated α-synuclein using quiescent seed amplification assay for the diagnosis of Parkinson’s disease

**DOI:** 10.1186/s40035-024-00426-9

**Published:** 2024-07-24

**Authors:** Hengxu Mao, Yaoyun Kuang, Du Feng, Xiang Chen, Lin Lu, Wencheng Xia, Tingting Gan, Weimeng Huang, Wenyuan Guo, Hancun Yi, Yirong Yang, Zhuohua Wu, Wei Dai, Hui Sun, Jieyuan Wu, Rui Zhang, Shenqing Zhang, Xiuli Lin, Yuxuan Yong, Xinling Yang, Hongyan Li, Wenjun Wu, Xiaoyun Huang, Zhaoxiang Bian, Hoi Leong Xavier Wong, Xin-Lu Wang, Michael Poppell, Yi Ren, Cong Liu, Wen-Quan Zou, Shengdi Chen, Ping-Yi Xu

**Affiliations:** 1 Department of Neurology, the First Affiliated Hospital of Guangzhou Medical University, Guangzhou, 510120 China; 2 School of Basic Medical Science, Guangzhou Medical University, Guangzhou, 511436 China; 3 Interdisciplinary Research Center on Biology and Chemistry, Shanghai Institute of Organic Chemistry, Chinese Academy of Sciences, Shanghai, 201210 China; 4 Deptartment of Neurology, The First Affiliated Hospital of Gannan Medical University, Ganzhou, 341000 China; 5 Institute of Neurology, Jiangxi Academy of Medical Clinical Sciences, The First Affiliated Hospital, Jiangxi Medical College, Nanchang University, Nanchang, 330006 China; 6 Bio-X Institutes, Key Laboratory for the Genetics of Developmental and Neuropsychiatric Disorders (Ministry of Education), Shanghai Jiao Tong University, Shanghai, 200030 China; 7 The Second Affiliated Hospital of Xinjiang Medical University, Urumqi, 830054 China; 8 Department of Neurology, Xinjiang Uygur Autonomous Region People’s Hospital, Urumqi, 830054 China; 9 Department of Neurology, Zhongshan City People’s Hospital, Zhongshan, 528400 China; 10 Dongguan Songshan Lake Central Hospital, Dongguan, 523000 China; 11 Jockey Club School of Chinese Medicine, Baptist University Road, Hong Kong, 999077 China; 12 Department of Nuclear Medicine, the First Affiliated Hospital of Guangzhou Medical University, Guangzhou, 510120 China; 13 Department of Biomedical Sciences, College of Medicine, Florida State University, Tallahassee, 32306 USA; 14 State Key Laboratory of Chemical Biology, Shanghai Institute of Organic Chemistry, Chinese Academy of Sciences, Shanghai, 200032 China; 15 Department of Neurology, Rui Jin Hospital Affiliated to Shanghai Jiao Tong University School of Medicine, Shanghai, 200025 China

**Keywords:** α-Synuclein, Seed amplification assay, Quiescent SAA, Parkinson's disease, Seeding activity

## Abstract

**Background:**

Seed amplification assays (SAA) enable the amplification of pathological misfolded proteins, including α-synuclein (αSyn), in both tissue homogenates and body fluids of Parkinson’s disease (PD) patients. SAA involves repeated cycles of shaking or sonication coupled with incubation periods. However, this amplification scheme has limitations in tracking protein propagation due to repeated fragmentation.

**Methods:**

We introduced a modified form of SAA, known as Quiescent SAA (QSAA), and evaluated biopsy and autopsy samples from individuals clinically diagnosed with PD and those without synucleinopathies (control group). Brain biopsy samples were obtained from 14 PD patients and 6 controls without synucleinopathies. Additionally, skin samples were collected from 214 PD patients and 208 control subjects. Data were analyzed from April 2019 to May 2023.

**Results:**

QSAA successfully amplified αSyn aggregates in brain tissue sections from mice inoculated with pre-formed fibrils. In the skin samples from 214 PD cases and 208 non-PD cases, QSAA demonstrated high sensitivity (90.2%) and specificity (91.4%) in differentiating between PD and non-PD cases. Notably, more αSyn aggregates were detected by QSAA compared to immunofluorescence with the pS129-αSyn antibody in consecutive slices of both brain and skin samples.

**Conclusion:**

We introduced the new QSAA method tailored for in situ amplification of αSyn aggregates in brain and skin samples while maintaining tissue integrity, providing a streamlined approach to diagnosing PD with individual variability. The integration of seeding activities with the location of deposition of αSyn seeds advances our understanding of the mechanism underlying αSyn misfolding in PD.

**Supplementary Information:**

The online version contains supplementary material available at 10.1186/s40035-024-00426-9.

## Introduction

Parkinson's disease (PD), together with multiple system atrophy (MSA) and dementia with Lewy bodies (DLB), forms the group of synucleinopathies that are characterized by accumulation of abnormal α-synuclein (αSyn) in the brain [1]. The formation and accumulation of misfolded αSyn aggregates as Lewy bodies (LB) or glial cytoplasm inclusions (GCIs) in the brain is a molecular hallmark of this group of disease [2]. A large body of evidence has confirmed that pathological αSyn aggregates possess prion-like properties for the recruitment and conversion of free monomers, and can undergo cell-to-cell transmission between the central and the peripheral nervous systems [3–5].

Remarkably, one of the recent revolutionary developments is to apply ultrasensitive in vitro amplification techniques including real-time quaking-induced conversion (RT-QuIC) and protein misfolding cyclic amplification (PMCA) to detect pathological αSyn aggregates in the cerebrospinal fluids (CSF) and peripheral tissues [6–11]. By taking advantage of the prion-like seeding activity of misfolded αSyn, these techniques enable exponential replication of a minimal amount of pathological αSyn from these specimens in vitro. Indeed, several lines of evidence have also revealed that αSyn seed amplification assays (SAAs) could be a robust tool for early disease detection and disease progression monitoring in clinical and research settings [12–15]. However, both RT-QuIC and PMCA require either shaking or sonication procedure, which are thought to fragment larger αSyn aggregates into smaller ones. This fragmentation process enables the αSyn seeds to undergo exponential amplification during incubation with excess recombinant monomeric αSyn [16–19].

Building upon the principles of classic SAA, we have developed a novel method termed Quiescent SAA (QSAA), suitable for both brain homogenates and tissue sections (in situ). QSAA eliminates the need for shaking or sonication, requiring only a temperature-controlled fluorescence reader, while in situ QSAA necessitates a temperature-controlled incubator and a fluorescence microscope. Our QSAA method exhibited sensitivity and specificity levels exceeding 90% in differentiating between PD and non-PD cases, evident in both brain and skin tissue sections in this study. The development of this sensitive assay for detecting pathological αSyn aggregates, both within and beyond the brain, provides further insights into the spatial pathological characteristics of misfolded proteins in tissue sections.

## Materials and methods

### Generation of αSyn monomers and pre-formed fibrils (PFFs)

Purification of human/mouse full-length αSyn (1–140 amino acids) was performed as described previously with minor modifications [20]. In brief, BL21 (DE3) *E. coli* strain (TransGen, Beijing, China) was transformed with a plasmid expressing full-length *SNCA* (*Homo sapiens*) or *Snca* (*Mus musculus*). The culture medium was shaken at 230 rpm overnight until optical density reached 0.8 at 600 nm, followed by induction with 1.0 mM isopropyl β-D-thiogalactoside for 4 h. After centrifugation at 8000× *g* for 10 min at 4°C, the bacterial pellets were resuspended in high-salt buffer containing 10 mM Tris-HCl (pH 7.5), 500 mM NaCl, 1 mM EDTA, 1 mM PMSF, and protease inhibitors, and then sonicated on ice. The lysate was boiled for 10 min, followed by ultracentrifugation (Beckman, Brea, CA) at 20,000× *g* for 30 min at 4°C. After precipitation with 45% saturated ammonium sulfate (AS), the proteins were resuspended with a buffer containing 10 mM Tris-HCl, pH 7.5, and 50 mM NaCl. The pH of the solution was then adjusted to pH 3.0 using HCl. The proteins were removed by centrifugation and then the pH was adjusted back to pH 7.5 with NaOH. The proteins were then purified by HiTrap column (GE Healthcare, Anaheim, CA), and dialyzed using 10 mM Tris-buffer (pH 7.5) containing 50 mM NaCl before being filtered through a 100-kDa Amicon Ultra filter (Millipore, Temecula, CA).

The purified protein was used to generate αSyn PFFs according to the previous protocol [21]. Purified human/mouse αSyn was diluted in 10 mM Tris (pH 7.5) containing 50 mM NaCl, followed by shaking (Eppendorf, Hamburg, Germany) at 1000 rpm for 5–7 days to generate αSyn PFFs. Afterward, the αSyn PFFs were probe-sonicated at 20% power for a total of 60 pulses (1-s on and 1-s off). Endotoxin levels in αSyn monomers and PFFs were determined using an Endotoxin Detection kit (Bioendo Technology, Xiamen, China).

Human recombinant β-synuclein (βSyn) PFFs (Type 1) (Cat# SPR-457), human recombinant γ-synuclein (γSyn) PFFs (Type 1) (Cat# SPR-459), and human synthetic amyloid β 1-42 (Aβ_1-42_) PFFs (Cat# SPR-487) were obtained from StressMarq Biosciences (British Columbia, Canada). Additionally, human recombinant tau PFF variants ζ306 (2R) and K19CFh (3R) were from GenScript (Piscataway, NJ).

### Transmission electron microscopy (TEM)

For TEM, a 10 μl volume of 0.1 mg/ml PFFs or amplification products was loaded onto freshly glow-discharged 400 mesh carbon-coated copper grids (Zhongjingkeyi Technology, Beijing, China) for 2 min as previously described [22]. The grid was quickly rinsed with three drops of 50 mM Tris-HCI (pH 7.5) and then continuously floated on two drops of 0.75% uranium formate (Solarbio, Beijing, China). The grids were observed using a CM 120 transmission electron microscope (PHILIPS, Eindhoven, The Netherlands) using an acceleration voltage of 80–120 kV.

### Animals and stereotaxic injections

All animal experiments were conducted in accordance with relevant ethical regulations for animal testing and research and were approved by the Animal Care and Use Committee of Guangzhou Medical University. Male C57BL/6 mice aged 10–12 weeks (Guangzhou Medical University, Guangzhou, China) were kept under circadian rhythm and had free access to food and water. Then, the mice were subjected to unilateral cortical, hippocampal, and striatal injections under general isoflurane anesthesia. Biefly, 1 μl of mPFFs, mouse αSyn monomer, or PBS was injected into the unilateral cortex (AP, 0.0 mm; ML, −1.5 mm; DV, −1.6 mm), hippocampus (ML, −1.7 mm; AP, −2.5 mm; DV, −1.8 mm), and substantia nigra (ML, −1.0 mm; AP, −3.08 mm; DV, −4.5 mm) at an infusion rate of 1 μl/min using a stereotactic alignment system (Kopf Instruments, Tujunga, CA). Following the injection, the Hamilton syringe was kept in situ for an additional 5 min before the needle was removed. At all stages of the study, the researchers were blind to the experimental conditions. For further analysis, mice were transcardially perfused under isoflurane anesthesia by ice-cold PBS 6 months after the stereotaxic injections. The whole brain was collected for subsequent slicing and in situ amplification of αSyn aggregates.

### Brain specimens

Fourteen subjects with PD combined with large intracranial hematoma and six subjects with epilepsy were recruited from the First Affiliated Hospital of Guangzhou Medical University during 2003-2022 based on a definite neuropathological diagnosis [23, 24]. Brain biopsy samples were collected with informed consent from patients and approval by the Ethics Committee of First Affiliated Hospital of Guangzhou Medical University (Medical Research Ethics, MRE. No.155). A 3.0 × 3.0 × 3.0 mm^3^ sample from the area surrounding the lesion was collected for biopsy. Pathological specimens for patients with cerebral hemorrhage were exclusively obtained from the basal ganglia region, whereas for epilepsy cases, biopsies were taken from the layer closest to the cortex. All tissues were frozen at −80°C before analysis. Experimental analysis of these samples was carried out by investigators who were blinded to the patient's diagnosis. Two autopsy brain samples, one from a PD patient (Braak stage III, male, 77 years old) and the other from a non-PD control (male, 80 years old), were ethically recruited at the First Affiliated Hospital of Guangzhou Medical University from 2005 to 2012. Brains were collected from the donors within 12 h after death. The brain was sagittally sectioned, and the left hemisphere was fixed in 10% buffered formaldehyde, while the right hemisphere was coronally sectioned and frozen at −80°C. The frozen tissue from the striatum was chosen for subsequent slicing and in situ amplification, without undergoing any pretreatment.

For brain homogenates, brain samples were processed to achieve a 10% (*w*/*v*) homogenate using a tissue homogenizer (Xinzhi, Ningbo, China) with 5-mm zirconia beads. The buffer was PBS with 1 mmol/l EDTA, 150 mmol/l NaCl, 0.5% Triton X-100, and a comprehensive protease inhibitor cocktail from Roche (Basel, Switzerland). The samples underwent 5 homogenization cycles, each comprising 1 min of grinding and 3 min of cooling at 4°C. After homogenization, brief centrifugation at 3000× *g* for 5 min was performed and the supernatant was collected. For further experiments, the homogenate was diluted 1:10,000 using PBS to achieve the desired working strength.

For brain slices, frozen brain samples without any pre-treatment were embedded in the optimal cutting temperature (OCT) compound, sectioned into 14-μm-thick slices and mounted onto 15-mm-diameter glass slides.

### Skin biopsy

A total of 422 biopsy skin samples were collected, including 214 from subjects receiving a clinical diagnosis of probable PD based on the international diagnostic criteria [25], and 208 from controls with other clinical diagnoses, such as 28 with progressive supranuclear palsy (PSP), 30 cases of essential tremor (ET), 35 cases of Alzheimer's disease (AD), 43 cases of epilepsy, 6 cases of viral encephalitis (VE), and 66 healthy controls (HC). All participants were included from the Department of Neurology of the First Affiliated Hospital of Guangzhou Medical University during 2016–2022. After reviewing the clinical history and neurological assessments conducted by specialists in neurodegenerative disorders, the patients underwent relevant diagnostic tests, including MRI, ^18^F-AV133 SPECT, or CSF analysis. Subsequently, employing the most recent recommended criteria for AD, PSP, ET, and VE, a final diagnosis was established for each patient [26–28]. Biopsy tissues from the skin were collected with informed consent from patients and approval by the Ethics Committee of the First Affiliated Hospital of Guangzhou Medical University (No. EC-2016-005(XJS)). Using a ring drill extractor (USHIO, Tokyo, Japan), skin samples approximately 3.0 × 3.0 × 3.0 mm^3^ in size, containing the epidermis, dermis, and fat layer, were obtained by punch biopsy from the posterior cervical site. The biopsy specimens were divided into two halves perpendicularly, and both halves were blindly coded and stored at –80°C. For further analysis, skin samples, frozen without pre-treatment, were embedded in OCT compound, sliced into 14-μm-thick sections, and then affixed onto round glass slides with a diameter of 15 mm. Experimental analysis of these samples was carried out by investigators who were blinded to the patient diagnoses.

### SAA

The reaction buffer contained 100 mM phosphate buffer (pH 7.5–8.0), 40 μM Thioflavin T (ThT), 50 mM NaCl, and 0.5 mg/ml human αSyn monomer. A black 96-well plate with a clear bottom (Thermo Fisher Scientific, Waltham, MA) was added with 95 μl of reaction buffer and a 2-mm silica bead (Thermo Fisher Scientific) in each well. Reactions were seeded with 5 μl of the indicated amount of hPFFs (diluted from 1 mg/ml of hPFFs) or 1:10,000 diluted brain homogenates to a final reaction volume of 100 μl. The plates were incubated in a SpectraMax iD5 plate reader (Molecular Device, San Jose, CA) at 37°C for 96 h with intermittent shaking cycles (high speed, double orbital, shaking for 1 min followed by 14 min rest). ThT fluorescence (450 ± 10 nm excitation and 490 ± 10 nm emission; bottom read) was recorded at 15-min intervals in the plate reader. The resulting curves were used for the analysis of the lag phase and apparent growth rate. A sample was designated as positive if, within 96 h, at least 2 out of 3 technical replicates surpassed the defined cut-off threshold, which was established as the background fluorescence plus 5 standard deviations—equating to roughly 8.6% of the maximal fluorescence value observed.

### QSAA

The diluted PFFs or brain homogenates were added to an amplification buffer containing 1 mg/ml αSyn monomers, 10% *w*/*v* AS (Sigma-Aldrich, St. Louis, MA), 40 μM ThT, 50 mM NaCl, and 10 mM Tris-HCl (pH 7.5) in a total volume of 20 µl. The mixture was then moved into a 96-well polypropylene PCR plate and topped with 20 μl of paraffin oil to prevent evaporation of the liquid. The plate was sealed with sealing film (BioTss, Beijing, China) and loaded into a real-time PCR instrument. The plate was incubated at 40°C to 90°C based on experimental requirements without agitation. The FAM channel (Excitation: 452–486 nm, Emission: 505–545 nm) was used to detect the ThT fluorescence intensity over time. The resulting curves were used for the analysis of lag time and apparent growth rate. For further analysis, the fluorescence intensity readings were transferred to GraphPad Prism version 9, where the data were normalized to the maximum fluorescence. A sample was considered positive if, within an 18-h timeframe, the fluorescence in at least 2 out of 3 technical replicates surpassed the established threshold. This threshold was established at a level equal to the background fluorescence plus 5 standard deviations. After the incubation, 1.5 μl of the amplified product was spotted onto a slide and photographed using the fluorescein isothiocyanate (FITC) fluorescence channel when the droplet was comparably dry. For quantitative analysis, the relative area of the amplified products exhibiting a strong fluorescence signal was calculated using the ImageJ software (Madison, WI).

### In situ QSAA

Brain or skin slices were placed in 24-well plates with a sealing film (Thermo Fisher) and incubated with buffer containing 1 mg/ml mouse monomer (MM), 10% AS, 40 μM ThT, 50 mM NaCl, and 10 mM Tris-HCl (pH 7.5), at a total volume of 300 µl per well, at 70℃ for 12–24 h. Fluorescence images were collected in the FITC channel through a fluorescence microscope after 3 gentle washes in PBS (5 min/wash). Detection of aggregated ThT fluorescence signals in at least 2 out of 3 replicates was defined as a positive result. To obtain high-resolution fluorescent microscopic images of the amplified products in brain tissues, we placed the slide on a motorized stage and captured images at 20× and 63× magnifications using an SP8 Leica scanning confocal microscope. Specifically, we acquired a 400 μm-thick z-stack with a 0.5-μm step size in the FITC channel to visualize the QSAA amplification products.

### Immunofluorescence

Brain tissues were sliced into 14-µm sections and then fixed in 4% paraformaldehyde. The fixed samples were then immersed in PBS containing 0.01% Triton X-100 and 5% BSA for 1 h at room temperature (RT). After incubation with anti-p-syn (pS129 αSyn) (CST, Danvers, MA, No. 51253, 1:500) or anti-aggregated αSyn antibody (Abcam, Cambridge, UK, MJFR14-6-4-2, 1:500) overnight at 4°C, the sections were washed with PBS and incubated with Alexa Fluor 488-labeled secondary antibody (Thermo Fisher, 1:500 dilution) for 1 h at RT. For skin double immunofluorescence, 14-µm-thick sections were incubated overnight at room temperature with a mix of anti-PGP9.5 (Abcam, Cat# ab8189, 1:200) and anti-p-syn (pS129 αSyn) (CST, Cat# 51253, 1:200) followed by Cy3-conjugated goat anti-mouse or Alexa-488-conjugated goat anti-rabbit antibody (1:500). Images were obtained with a fluorescence microscope (NIKON ECLIPSE 3000, Tokyo, Japan). Phosphorylated αSyn was identified by the pS129 antibody, and nuclei were identified with DAPI.

### ^18^F-AV133 PET and CT image processing

The [^18^F]-DTBZ radiotracer (^18^F-AV133) was prepared from aqueous [^18^F]-fluoride. Subjects were administered with D6-[^18^F] FP-(+)-DTBZ intravenously at 10.0 mCi ±10% and rested for 55 min in a quiet, light-free room. Brain scans were acquired over approximately 60–70 min after completion of dynamic drug administration. Florbenazine images were spatially normalized to a standard atlas using an ^18^F-AV133 PET template. Atlas-based volumes of interest were applied for target areas (caudate, anterior and posterior putamen, and total striatum). The ratio of tracer activity in the target volumes of interest relative to the occipital cortex as a reference region was calculated to obtain the standard uptake value ratio.

### Statistical analysis

All statistical analyses were conducted with the GraphPad Prism software (San Diego, CA). Experimental data were analyzed using the two-tailed independent Student's* t*-test for comparing two groups and one-way analysis of variance (ANOVA) followed by Tukey's post-hoc test for comparison among three or more groups. Unless otherwise stated, data are presented as mean ± SD. *P* < 0.05 was considered statistically significant.

## Results

### Generation of a new version of SAA without shaking or sonication

**Fig. 1:**
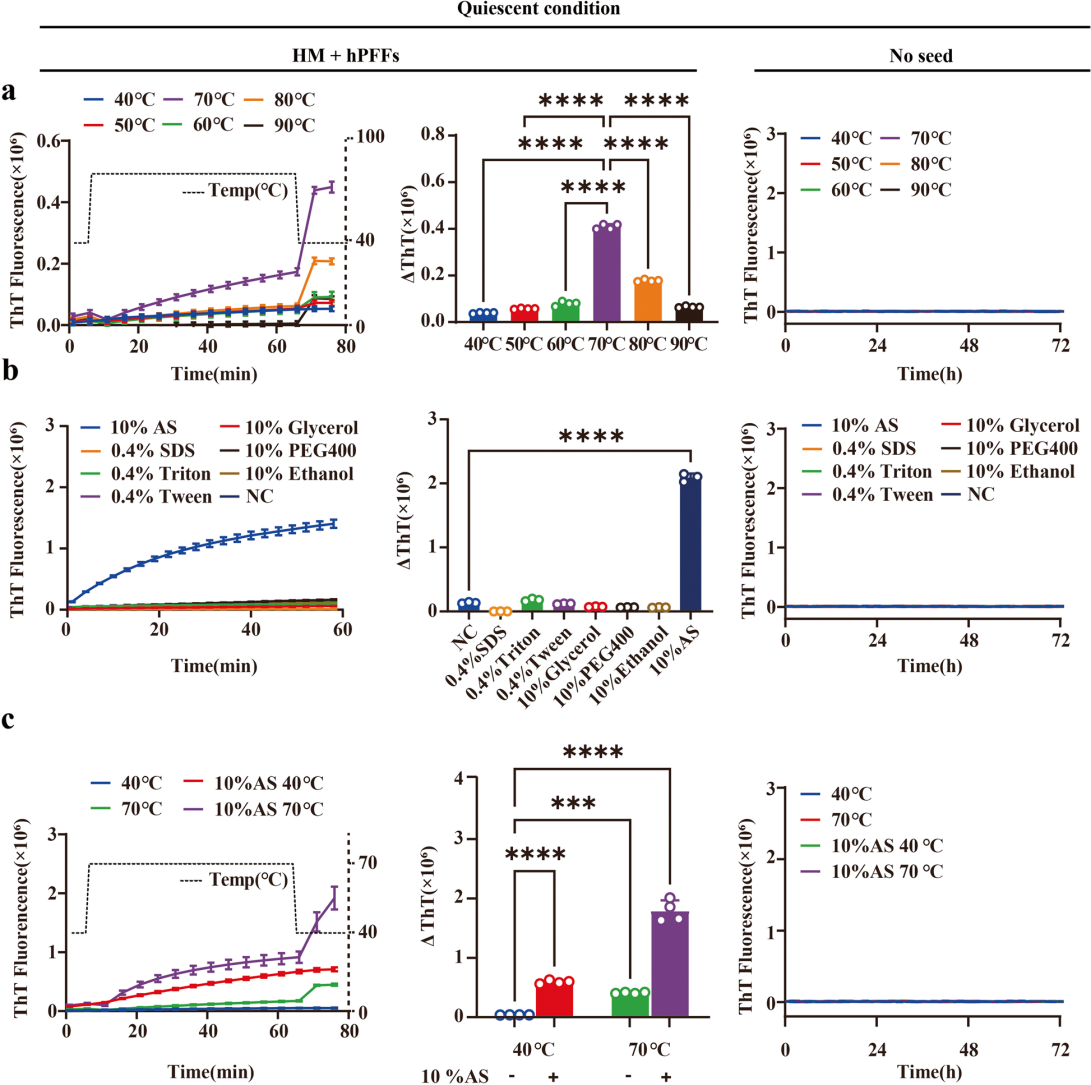
QSAA of human PFFs (hPFFs). The kinetics of fibril elongation was measured over time. The changes of ThT values (ΔThT) after quiescent incubation were quantified. **a** QSAA reaction at different temperatures. The optimum reaction temperature for HM + hPFFs was 70°C. In control experiments conducted in the absence of PFFs (*n* = 4), none of the incubation conditions resulted in a signal response. A temperature regression was conducted following amplification to correct for the impact of varying temperatures on ThT fluorescence. **b** QSAA reaction in the presence of different additives (10% AS, 10% ethanol, 10% glycerol, 10% PEG-400, 0.4% SDS, 0.4% Triton X-100, or 0.4% Tween-20). In control experiments conducted in the absence of PFFs (*n* = 4), none of the incubation conditions resulted in a signal response. **c** QSAA reaction at 40°C or 70°C in the presence of 10% AS. In control experiments conducted in the absence of PFFs (*n* = 4), none of the incubation conditions resulted in a signal response. All reaction conditions with seeds were supplemented with 0.01 mg/ml hPFFs in 1 μl. A temperature regression was conducted following amplification to correct for the impact of varying temperatures on ThT fluorescence. All data are presented as mean ± SD. *P < 0.05, **P < 0.01, ***P < 0.001, ****P < 0.0001 by one-way analysis of variance (ANOVA) followed by Tukey’s multiple comparison test

The pathological αSyn, which is expected to be present in an aggregated form, can act as seeds to spontaneously recruit soluble monomers for sef-replication [29, 30]. Several factors can affect the aggregation of αSyn, such as pH, temperature, metal ions, lipids, environmental toxins, molecular chaperones, inflammation, and genetic factors [31, 32]. To confirm that the quiescent conditions favor the elongation of PFFs generated from recombinant αSyn rather than nucleation of monomeric species [33], we modified the amplification mode by removing the shaking steps that might lead to the formation of spontaneous aggregates (αSyn_spon_) [34]. Our quiescent incubation buffer for the Thioflavin T (ThT) assay contained 0.01 mg/ml PFFs, 1.0 mg/ml monomer, 40 μM ThT, 50 mM NaCl, and 10 mM Tris-HCl pH 7.5. In addition, thermal intervention may increase the frequency of molecular collisions between seed and substrate to accelerate polymerization, making it more prone to fibrillation [35]. Therefore, different incubation temperatures (40°C, 50°C, 60°C, 70°C, 80°C and 90°C) were used to explore the effect of elevated temperature on fibrillation of αSyn under quiescent conditions. Consistent with previous studies, rapid amplification of αSyn occurred efficiently at all tested temperatures [36]. The optimal reaction temperature for the quiescent incubation system was 70°C in both the human monomer (HM) + human PFFs (hPFFs) and the MM + mouse PFFs (mPFFs) groups without any formation of αSyn_spon_ (Fig. 1a, Fig. S1a). Next, we tested the effect of different additives (10% *w*/*v* AS, 10% ethanol, 10% glycerol, 10% polyethylene glycol (PEG 400), 0.4% SDS, 0.4% Triton X-100, or 0.4% Tween-20) on αSyn elongation in vitro. The presence of 10% AS at 40°C resulted in ThT readings dozens of times higher as compared to the control group in both HM + hPFFs and MM + mPFFs without the generation of αSyn_spon_, while this phenomenon was not observed in other groups, indicating that the presence of 10% AS is an effective factor to enhance αSyn elongation (Fig. 1b, Fig. S1b).

To further verify the effect of 10% AS on the seeding activity of PFFs under different temperatures, the elongation rates of αSyn seeds at two thermal conditions (40°C, 70°C) were explored in the quiescent incubation system (0.01 mg/ml PFFs, 1.0 mg/ml monomer, 10% AS, 40 μM ThT, 50 mM NaCl, and 10 mM Tris-HCl pH 7.5). As expected, the presence of 10% AS significantly increased the efficiency of αSyn fibril elongation at both temperatures tested compared to the non-AS control group. Moreover, the increase of temperature from 40°C to 70°C in the presence of 10% AS led to a more significant increase in the total fluorescence in both groups (Fig. 1c, Fig. S1c). Importantly, in the absence of seeds, no αSyn aggregates were formed at either temperature tested within the period designed, with the addition of AS in the incubation system. Although further increase of the reaction temperature accelerated the reaction (data not shown), to avoid spontaneous conversion of αSyn monomers into αSyn_spon_ at higher temperatures, in subsequent experiments the QSAA was performed at amplification temperature of 70°C in buffer condition containing 1.0 mg/ml monomer, 10% AS, 40 μM ThT, 50 mM NaCl, and 10 mM Tris-HCl pH 7.5.

### Specificity and sensitivity of QSAA with PFFs

**Fig. 2:**
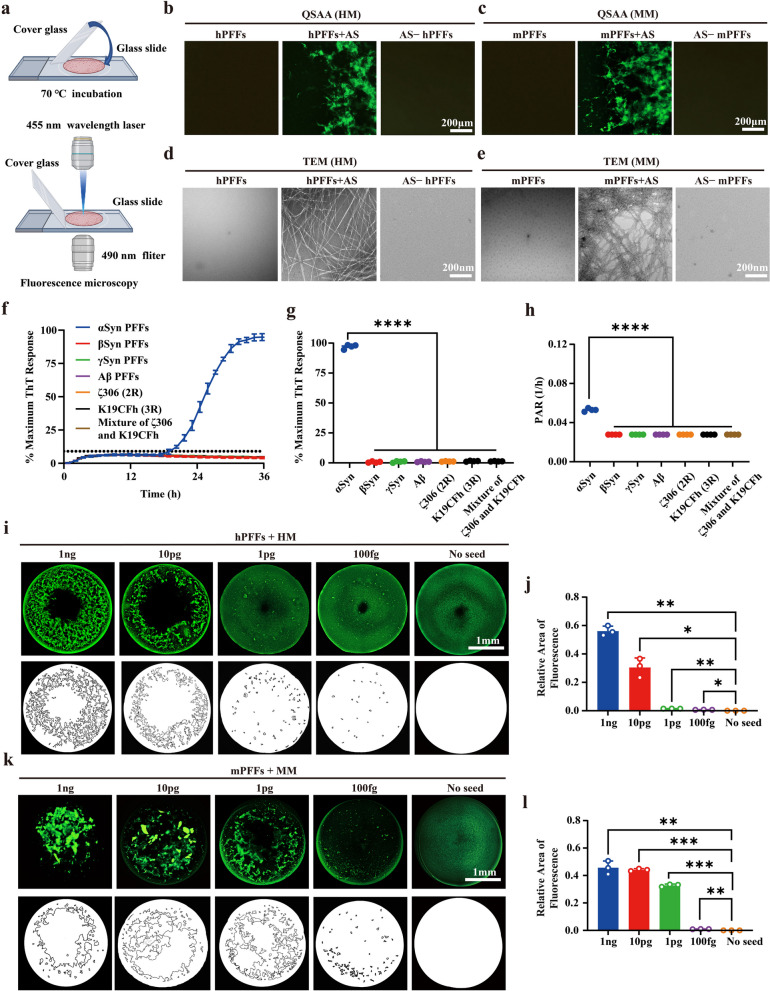
The detection limit of QSAA. **a** Schematic of the detection of quiescent amplification products by fluorescence. **b**, **c** Direct observation of amplification products under a fluorescence microscope. hPFFs/mPFFs (0.01 μg/ml) were incubated with 1 mg/ml human monomer (HM)/ mouse monomer (MM) and 40 μM ThT. QSAA was carried out at 70℃ for 24 h in the presence or absence of 10% AS. **d**, **e** TEM was performed to evaluate the fibrillar structure of the amplification products in (**b**, **c**). **f** QSAA using different seeds (100 fg αSyn hPFFs, 100 pg βSyn PFFs, 100 pg γSyn PFFs, 100 pg Aβ PFFs, 100 pg ζ306 (2R) PFFs, 100 pg K19CFh (3R) PFFs, 100 pg mixture of ζ306 PFFs and K19CFh PFFs). Data are presented as mean ± SD (*n* = 4). **g** Normalized maximum ThT fluorescence values for QSAA in (**f**). **h** The lag phase data from (**g**) were used to calculate the PAR (1/h) for both SAA and QSAA reactions. For reactions that did not produce ThT fluorescence surpassing the threshold (assigned a lag phase of 36 hours), the rate was established at 0.027. **i**, **k** Serial dilutions of the hPFFs/mPFFs (equivalent to 1 ng, 10 pg, 1 pg, and 100 fg PFFs) were added to the quiescent incubation system, together with 1 mg/ml HM/MM, 10 mM Tris-HCl pH 7.5, 50 mM NaCl, and 40 μM ThT in a total volume of 20 μl. Fluorescence images were collected at 24 h of QSAA reaction. Panels below the fluorescence images are schematic diagrams illustrating the amplification products. **j**, **l** Calculation of the relative fluorescence area of filamentous or lamellar amplification products within circular droplets. Data presented as mean ± SD (*n* = 3). *P < 0.05, **P < 0.01, ***P < 0.001, ****P < 0.0001 by one-way analysis of variance (ANOVA) followed by Tukey’s multiple comparison test

Eliminating the agitation cycle, αSyn was observed to undergo continuous amplification, serving as the foundation of the QSAA assay. Since the wavelength range of the FITC filter (Excitation: 470–480 nm, Emission: 510–530 nm) is similar to that of ThT fluorochrome which binds to the β-sheet structure of amyloid fibrils (Excitation: 450 nm, Emission: 495 nm), it seems feasible to directly observe the amplification products by fluorescence microscopy. At 24 h post-amplification, 1 μl of the amplified product from both hPFFs and mPFFs was spotted on the slide, and fluorescence was recorded in the FITC channel (Fig. 2a). Compared to the non-AS control group, many distinguishable green filaments were visible under the microscope in the AS group (Fig. 2b, c). Notably, there was no αSyn_spon_ formation under the quiescent conditions over 24 h, confirming the specificity of our QSAA. To further confirm the characteristics of these green filaments, the products were morphologically verified by TEM. Consistently, QSAA in the absence of AS resulted in no observable filaments after 24 h of incubation. αSyn PFFs per se also showed no filaments. However, in the presence of AS, a significant number of typical long unbranched amyloid fibrils were clearly visible under TEM (Fig. 2d, e). This indicates that QSAA not only accelerates protein aggregation but also maintains the typical β-sheet structure of the amplification products. In addition, directly visualizing the amyloid fibrils through fluorescence microscopy is an additional method to validate the amplification of αSyn.

To further determine the specificity of our QSAA, we next investigated the possible cross-seeding activity of QSAA. The hPFFs of other human-derived amyloid proteins including ζ306 (2R), K19CFh (3R), mixture of ζ306 and K19CFh, Aβ, βSyn and γSyn PFFs were used to seed αSyn. Consistently, no signal was detected in the presence of any of these amyloid PFFs, even at a higher concentration (100 pg), within 36 h (Fig. 2f–h). This outcome validates the specificity of the QSAA assay, showing no cross-seeding between αSyn monomers and other types of amyloid PFFs.

To explore the detection limit of QSAA, we performed serial dilutions for both hPFFs and mPFFs using Tris-buffer, pH 7.5. The number of distinguishable filaments decreased with lower concentrations of seeds (Fig. 2i–l). Even at the lowest concentrations of both hPFFs and mPFFs (equates to approximately 100 fg of aggregated protein), a small amount of fibrillar products could still be detected after 24 h of amplification, while no aggregates were seen in the group without PFF addition. In other words, the detection limit of PFFs in QSAA reached 100 fg. Interestingly, the difference in fibril production following QSAA becomes apparent when comparing hPFFs+HM to mPFFs+MM at lower seed concentrations. This variation in quantification likely stems from the quicker amplification rate of MM in comparison to HM, a phenomenon that has been observed in another study [37]. When amplified at very low concentrations such as 1 pg, hPFFs was unable to be amplified to a comparable amount of fibrils as mPFFs within a 24-h timeframe. This underscores the varying amplification efficiency among different species. Overall, these findings indicate that performing a single endpoint fluorescence reading in a separate fluorimeter enables the specific detection of αSyn seeding activity by QSAA with little, if any, loss of sensitivity.

### Comparison of QSAA and classic SAA using PFFs and brain homogenates of PD as seeds

**Fig. 3:**
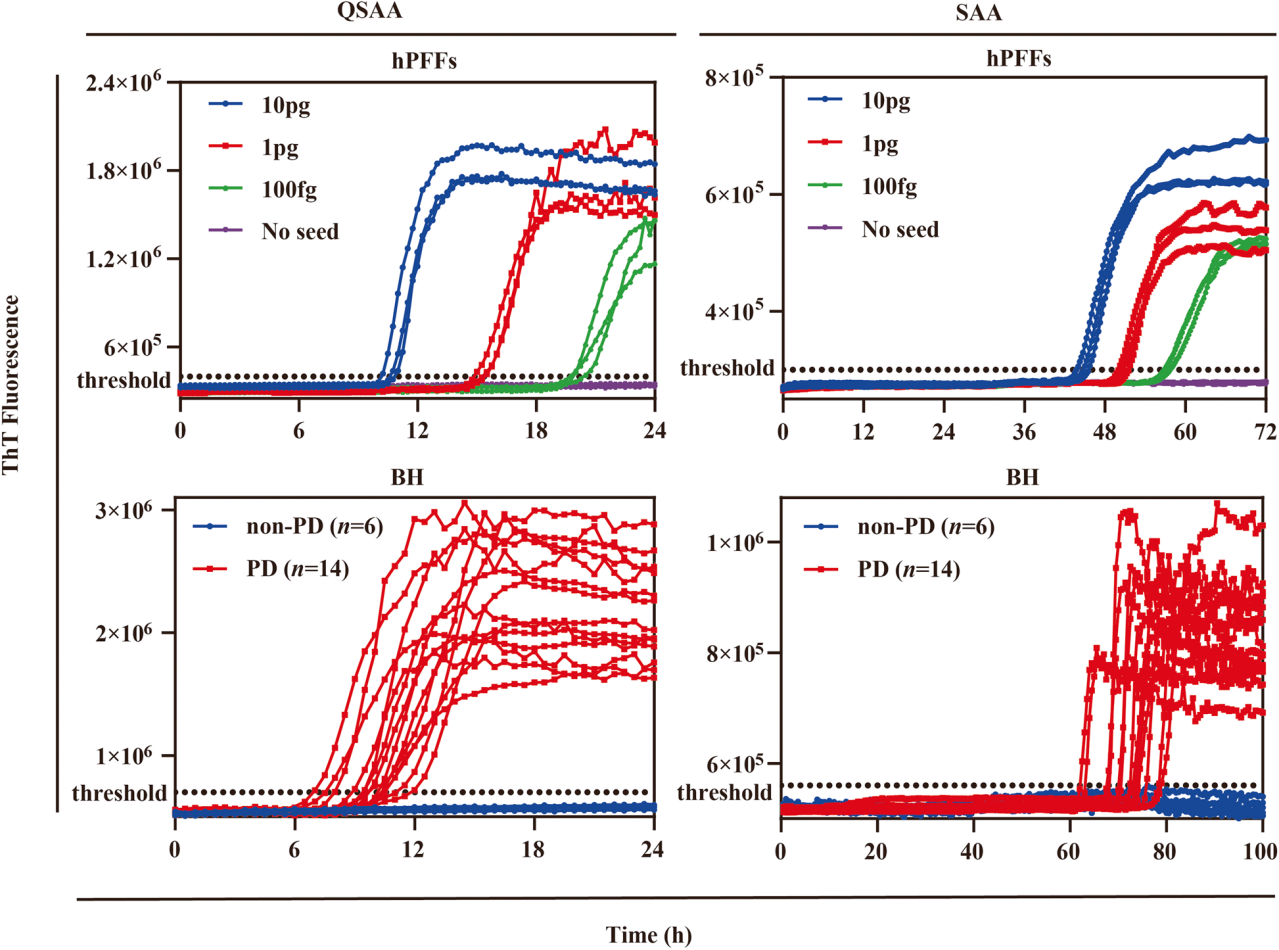
Detection of αSyn seeding activity of hPFFs and brain homogenates by SAA and QSAA. Upper: ThT fluorescence of SAA and QSAA reactions seeded with different concentrations of hPFFs (*n* = 3). In control-seeded reactions, neither the incubation of monomeric αSyn using SAA nor QSAA exhibited a significant influence on the signal response. Lower: ThT fluorescence in SAA and QSAA assays using biopsied brain homogenates from patients with PD (*n* = 14) and non-PD controls (*n* = 6) as seeds. Each point represents the mean of three technical replicates

To evaluate the amplification efficiency of QSAA, we performed a side-by-side comparison with RT-QuIC, the classic SAA with shaking. We first carried out comparative experiments using artificial seeds. Both the SAA and QSAA assays readily detected αSyn seeding activity using the same serial dilution of hPFFs. This was evidenced by the enhanced ThT fluorescence observed with hPFFs at all dilutions, indicating no difference in the detection limit between the two methods (Fig. 3).

To further evaluate the diagnostic utility of the QSAA assay, biopsy brain samples from clinically confirmed PD patients (*n* = 14) and non-PD controls (*n* = 6) were tested blindly. Demographic and clinical information of the patients is listed in Additional file 1: Table S1. The brain homogenates diluted at 1:10,000 were analyzed by SAA and QSAA simultaneously. αSyn seeding activity was found to be positive in 14 out of the 14 PD cases in both SAA and QSAA (Fig. 3). In contrast, no ThT-fluorescence signals were detected in controls in both assays (Fig. 3). Therefore, both methods had a sensitivity of 100% (14/14) and a specificity of 100% (6/6) (Table 1). Significantly, all samples generated fluorescence signals within 14 h in the QSAA assay, which completed at 24 h. In contrast, the SAA assay began to detect fluorescence signals at 72 h and completed at 96 h. In addition, the classic SAA and QSAA protocols consistently identified each PD patient and each non-PD control (Table S2). In addition, we obtained a Cohen's kappa value of 1.00 (95% CI:1.0) between SAA and QSAA, which indicates a strong consistency between the assays.

**Table 1:** Demographics and brain biopsy sample analysis by SAA and QSAA

	**PD (*n* = 14)**	**Non-PD (*n* = 6)**
Age (years), mean ± SD	65.86 ± 5.10	40.83 ± 6.15
Sex, male (female), *n*	9 (5)	3 (3)
Disease duration (years), mean ± SD	7.71 ± 2.81	NA
Hoehn und Yahr stage, mean ± SD	3.21 ± 0.65	NA
αSyn SAA-positive in brain homogenates *n* (%)	14 (100%)	0 (0%)
αSyn QSAA-positive in brain homogenates, *n* (%)	14 (100%)	0 (0%)
IF (pS129)-positive in brain slices, *n* (%)	14 (100%)	0 (0%)
αSyn QSAA (in situ)-positive in brain slices, *n* (%)	14 (100%)	0 (0%)

*Abbreviation*: *SAA* Seed amplification assay, *QSAA* Quiescent seed amplification assay, *IF* Immunofluorescence, *NA* Not available

### Amplification of αSyn aggregates in biological PD samples by in situ QSAA

The advantage of QSAA over classic SAA is that the amplification reaction can be performed stably without shaking or sonication, thereby allowing the amplification to be done quiescently while keeping the tissue sections intact. Therefore, it is feasible to use QSAA to localize the pathological αSyn seeds in tissue samples in situ. To this end, mPFFs were injected ipsilaterally into the cortex, hippocampus, and substantia nigra of mice. Each injection delivered 2.5 μg, with 1 μl of mPFFs administered at each point. To identify the compound that may be responsible for in situ QSSA, we explored monomeric species described to affect αSyn aggregation kinetics. Results from in situ QSAA indicated that treatment of the mPFF-inoculated brain sections with 10% AS (which affected ionic strength) together with MM, successfully induced a robust amount of folding in pathological αSyn. On the contrary, when the samples were treated with 10% AS along with HM, or in the absence of AS, the positive signal was significantly lower (Fig. S2), confirming that both AS and MM are required for the success of quiescent amplification.

**Fig. 4:**
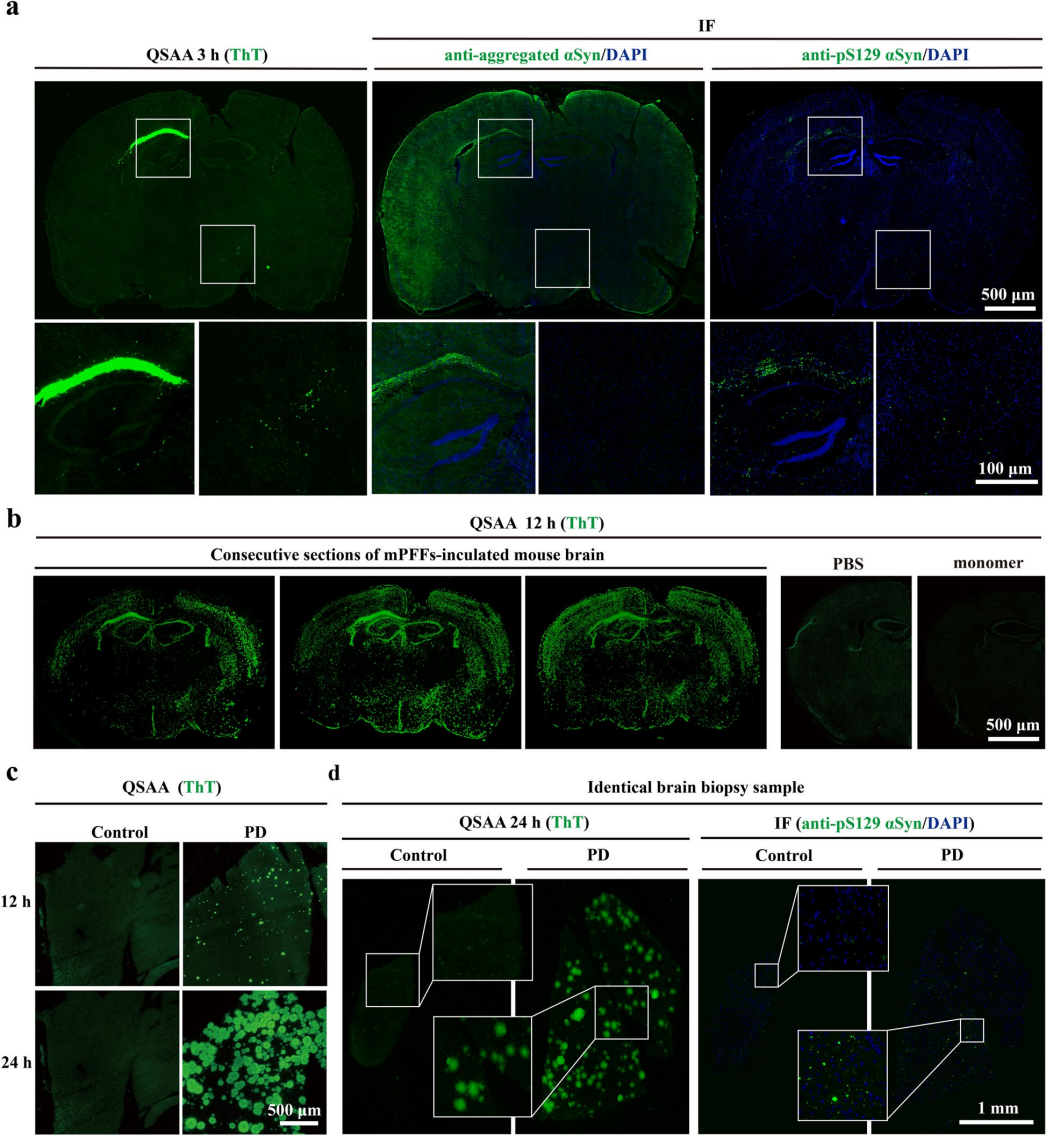
In situ amplification of αSyn aggregates in brain tissues. **a** Pathological αSyn was detected using both IF (pS129 and the anti-aggregated αSyn antibody) and QSAA in the mPFF-inoculated mouse brains (*n* = 6/group).Three consecutive sections were used for assessment. The zoomed captions were obtained with a 63× lens. **b** Representative images of three consecutive sections from a mPFF-inoculated mouse brain after QSAA for 12 h. No positive signal was detected in the PBS and monomer groups (*n* = 6/group). **c** Representative images of biopsy brain sections from PD patients and controls after QSAA for 12 h and 24 h. **d** Representative images of pathological αSyn detected using both QSAA and IF (pS129) in PD and control brains

To investigate the distinction between QSAA and immunohistochemistry in detecting pathological αSyn, three consecutive slides from a mouse that was inoculated with mPFFs were used for QSAA, immunohistochemical evaluation of pS129, and that of aggregated αSyn, respectively. In line with prior research, mPFF-induced αSyn pathology was observed in the brains of wild-type mice, as evidenced by both IF and QSAA, particularly prominent in the corpus callosum six months after injection (Fig. 4a) [38, 39]. Both QSAA and IF characterized pathological αSyn, with relatively low levels of αSyn detected by the anti-pS129 αSyn antibody and anti-aggregated αSyn antibody MJFR14-6-4-2 (Fig. 4a). While there was a clear overlap observed between pS129–αSyn and QSAA aggregates, there were also distinct QSAA aggregates present. This observation underscores both the consistency and novelty of QSAA in identifying pathological αSyn.

Another concern is the diffusion of the QSAA products. Indeed, secondary nucleation may occur during amplification even without shaking, which leads to the formation of smaller fibrils/oligomers that would be able to diffuse away from the initial seed, especially due to convection. To investigate seed displacement following secondary nucleation in the amplification process, we amplified the seeds using QSAA in three consecutive slices in a mPFF-inoculated mouse. Considering the relatively weak connection between new seeds generated by convection and the tissue, we performed three washes with PBS after amplification to remove nonspecific amplification signals. We observed that the locations of the amplification products in each brain slice were similar and appeared spotty after 12 h of incubation, showing no obvious difference in the distribution. No fluorescence signal was detected in the PBS- or monomeric αSyn-inoculated control mice (Fig. 4b). These observations indicate that diffusion may occur in QSAA, but it does not significantly affect interpretation of results. The post-amplification wash is a crucial step for removing non-specific amplification.

To enhance our understanding of the physical features of the amplified product, the structures of the plaques were visually verified by confocal microscopy (Fig. S3). These images depicted irregularly intertwined fibrils forming clumps in the mPFF-inoculated brain, surrounded by fine fibrils resembling thorns. Such structures suggest that the plaques are products of continuous amplification of amyloid-like fibrils.

The in situ QSAA was further used to investigate the aforementioned brain biopsy samples from PD patients and controls. During quiescent incubation of these biological samples, the spotty formations became enlarged from 12 h to 24 h of amplification (Fig. 4c). The αSyn seeding activity was present in 14 out of the 14 PD cases. Conversely, none of the control cases exhibited any ThT-fluorescence signal, highlighting the in situ QSAA with 100% sensitivity (14/14) and specificity (6/6) (Table 1). Moreover, all PD patients (14/14) tested positive using IF (Table 1), indicating that the sensitivity of in situ QSAA matched that of IF. Both histological staining and in situ QSAA in biopsy and autopsy samples consistently revealed similar distribution patterns across consecutive brain slides (Fig. 4d, Fig. S4). Although we noticed some overlap between pS129–αSyn and QSAA aggregates, QSAA produced more signals than pS129 immunohistochemistry. This indicates the potential existence of non-phosphorylated aggregates, suggesting that pS129-αSyn may not adequately reflect the extent of seeding activity. Technically, consecutive slides by cryosectioning (14 μm) may not adequately capture the complete profile of a cell as the average diameter of a neuronal soma in the cortical pyramidal layer does not exceed 10 μm [40]. Therefore, the consecutive slides may not be identical.

### Detection of αSyn aggregates in skin samples using in situ QSAA

**Fig. 5:**
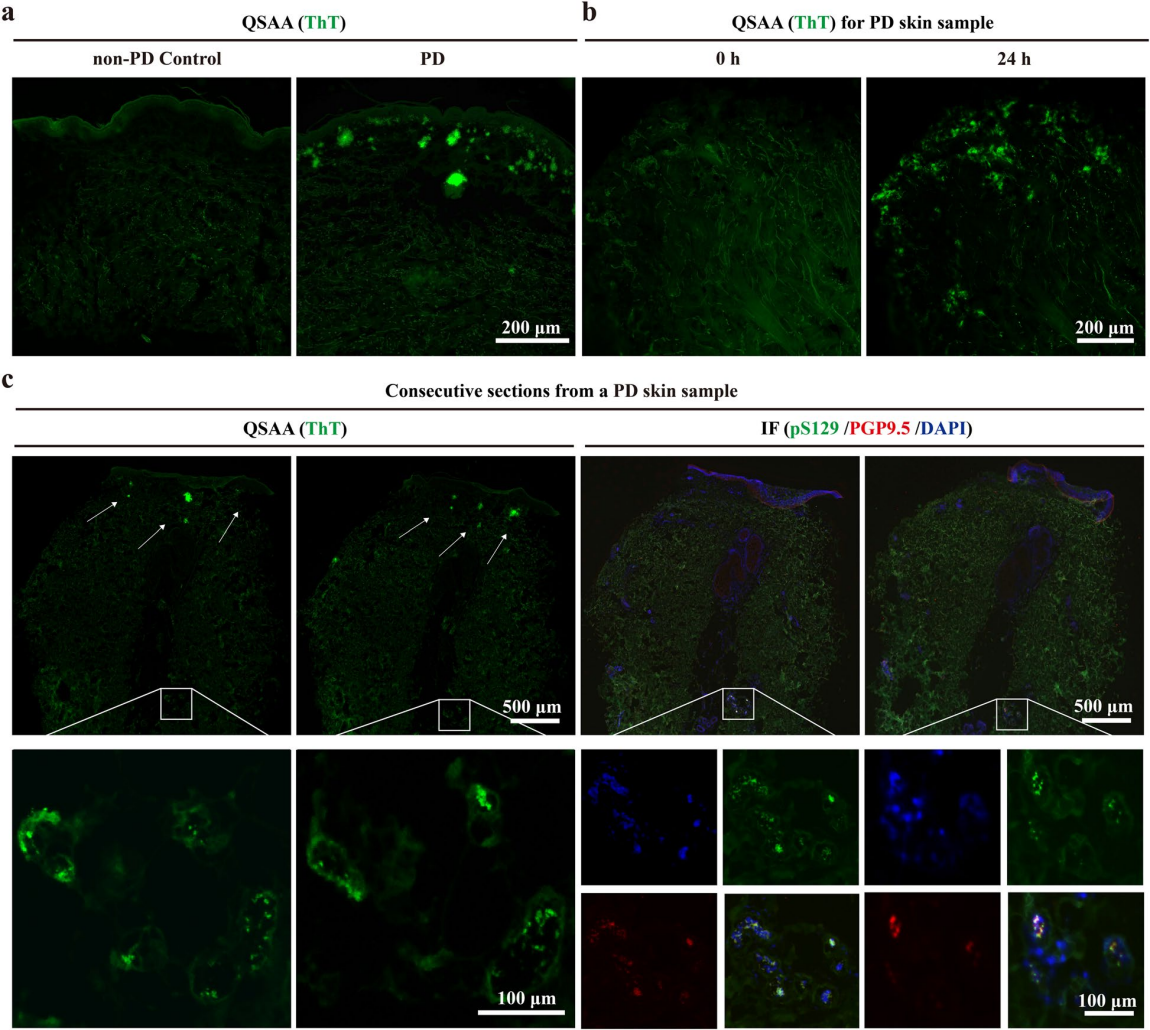
Detection of αSyn aggregates in skin samples using QSAA. **a**, **b** Formation of abnormal clusters of pathological αSyn was detected in skin samples from PD patients by QSAA. **c** Pathological αSyn was detected by both QSAA and IF (*green* pS129, *Red* PGP9.5) in consecutive sections from a PD skin sample. White arrows show the location of pathological αSyn detected by QSAA (green) signals

Several studies have shown that αSyn deposits within structures of the peripheral nervous system such as dermal nerve fibers, which provides proof-of-concept that skin αSyn aggregates may serve as a novel biomarker for antemortem diagnosis of PD [41–43]. We then tested whether in situ QSAA can be equally applied to skin samples obtained by punch biopsy from posterior cervical sites of PD patients. As expected, abnormal clusters of αSyn were shown in the dermis layer of patients with clinically diagnosed PD, as indicated by ThT signals after quiescent amplification for 24 h (Fig. 5a, b). In contrast, no ThT signals were found in the non-PD controls. We also conducted immunostaining for pS129 and PGP9.5 (a neuronal marker), and compared this signal with QSAA using consecutive sections from the same sample (Fig. 5c). Consistent with previous findings [44],in situ QSAA revealed αSyn aggregate distribution centered in the sub-epidermal layer and sweat glands, and co-localization of pS129 and PGP9.5 was observed in the sweat glands within the same skin sample.

To expand the applicability of QSAA, a total of 422 skin samples were analyzed, including 214 samples from clinically diagnosed PD patients and 208 samples collected from either healthy individuals or patients with other neurologic diseases. Analysis of skin samples obtained from PD patients revealed a higher seeding activity, with 193 out of 214 samples (90.19%) displaying positive signals in QSAA testing. In contrast, among the 208 control samples, 190 (91.35%) displayed negative signals in QSAA testing (Table 2).

**Table 2:** Demographics and skin tissue analysis by in situ QSAA

	**PD (*n* = 214)**	**Non-PD controls**^**a**^** (*n* = 208)**
Age (years), mean ± SD	63.73 ± 9.25	58.19 ± 9.98
Sex [Male (Female)], *n*	111(103)	115 (93)
Disease duration (years), mean ± SD	6.84 ± 3.86	NA
Hoehn und Yahr stage, mean ± SD	2.91 ± 0.72	NA
αSyn QSAA (in situ), positive (negative), *n*	193 (21)	18 (190)

*Abbreviation*: *QSAA* Quiescent seed amplification assay, *PD* Idiopathic Parkinson’s Disease, *NA* Not available

^a^Non-PD controls include 28 progressive supranuclear palsy, 30 essential tremor, 35 Alzheimer's disease, 43 epilepsy, 6 viral encephalitis, and 66 healthy controls

Subsequently, we randomly chose 18 QSAA-positive PD patients and two healthy controls for PET-CT AV133 scanning. The results confirmed a progressive asymmetric decrease in dopamine metabolism in the striatum of all PD patients, supporting the specificity of the assay for the diagnosis of PD (Fig. S5).

## Discussion

Based on the prion-like properties, several lines of evidence have demonstrated that SAA could serve as a valuable diagnostic tool for the detection of abnormal αSyn aggregates. A number of groups have successfully applied SAA assays on a variety of biological samples to detect the seeding activity of αSyn, by taking advantage of the intrinsic property of amyloid fibrils to self-template [45–52]. In this study, we developed an amplification technique QSAA that allowed in situ amplification of abnormal conformers of pathological αSyn aggregates in brain and skin sections from PD patients. This study showcased the ability of QSAA to amplify seeds within samples in situ, with the resulting amplification products being directly observable upon binding with ThT dye. These signals were easily discernible and unveiled notable disparities between PD patients and control subjects. The QSAA method is superior to the classic SAA due to its capability for in situ amplification, which allows for not only ultrasensitive detection but also precise localization of pathological αSyn within tissues. The QSAA method is a potential diagnostic tool for synucleinopathies in clinical settings.

A fundamental question is what factors accelerate αSyn fibrillation. The classic SAA system consists of αSyn aggregates (seeds), recombinant monomeric αSyn (substrates), mixed buffer, ThT fluorescent dye, pH, ionic strength, and other influencing factors such as temperature, sonication, cycled shaking, etc. All these together determine the efficiency and specificity of αSyn amplification. Uversky et al. found that an increase in temperature or a decrease in pH, is strongly correlated with enhanced formation of αSyn fibrils [53]. Munishkina et al. found that anions greatly accelerate αSyn fibrillation [54]. To achieve accurate diagnosis in a short time, we have developed the QSAA assay through four distinct steps: (1) raising the incubation temperature to 70°C; (2) implementing a quiescent mode for incubation; (3) using mouse αSyn monomers rather than human αSyn monomers, and (4) adding 10% AS to the incubation buffer. Our data demonstrated that the four modifications greatly accelerated the speed of αSyn aggregation. As indicated previously, mouse αSyn with A53T mutation dominates the growth kinetics and shows a much shorter lag phase than human αSyn and human A53T in fluorescence analysis [37]. Anions, especially SO^4-^, induce partial folding of αSyn at neutral pH, forming a critical amyloidogenic intermediate, which leads to a significant acceleration of the rate of fibrillation [54, 55]. In addition, elevating temperature significantly increases the frequency of molecular collisions, which may allow the amyloid fibrils to extend rapidly at quiescent conditions.

Amplifying target proteins in situ based on the seeding capacity provides additional information for the understanding of αSyn seeding in the brains of PD. Both SAA and QSAA are based on the prion-like seeding activity of αSyn. However, the in situ amplification assay allows the detection and localization of pathological αSyn in tissue sections, which would add new parameters such as spatiotemporal dynamics of pathological αSyn and visualize the morphological characteristics of the amplified products. There is also a possibility that the characterization of the size and the spatial distribution of amplified products may provide a unique signature for each PD patient, providing new insights into synucleinopathies.

There is no doubt that pS129 αSyn is a useful and relatively specific pathological marker of Lewy body disease (LBD). Benefiting from the amplification and signal enhancement features of SAA, QSAA demonstrates greater advantages compared to IF. As a novel form of SAA, QSAA demonstrates markedly increased sensitivity compared to pS129 in the detection of pathological αSyn [56, 57]. Moreover, because αSyn pS129 occurs subsequent to initial protein deposition and inhibits seeded fibril formation, αSyn aggregates may be formed early and are associated more with pathological propagation [58]. Additionally, QSAA assays are tailored to target specific pathologically misfolded αSyn aggregates associated with PD, reducing the risk of false positives compared to IF, which may exhibit cross-reactivity with other cellular components or proteins, potentially leading to misinterpretation [59, 60]. It is noteworthy that a small fraction (about 4%) of αSyn is phosphorylated at S129 in normal brains, but in LBD cases pS129 dramatically accumulates (>90% of αSyn), indicating that not all instances of LBD are exclusively comprised of phosphorylated αSyn [61]. Thus, the clinical value of pS129 αSyn as the only pathological marker is limited.

Collectively, our study demonstrated a new version of SAA for identifying the formation of abnormal clusters of αSyn in situ based on quiescent fibrillar elongation, which provides a practical way to distinguish PD cases from controls. The technique allows a comprehensive and multilayered understanding of PD pathogenesis that is fundamental to the development of novel therapeutic strategies.

## Conclusion

In this study, we developed a novel seed amplification assay called QSAA, designed for the in situ amplification of αSyn aggregates in brain and skin samples. Unlike established αSyn SAAs, which rely on shaking or sonication to significantly enhance αSyn fibril amplification, QSAA eliminates the necessity for cyclic fragmentation, offering a one-step approach to diagnosing PD with individual heterogeneity. This technique facilitates quiescent amplification of pathological αSyn, not only in brain homogenates but also in tissue sections (in situ), marking a milestone by directly correlating αSyn seeding activity with the spatial distribution of pathological αSyn in biological samples. Notably, QSAA has demonstrated high sensitivity (90.2%) and specificity (91.4%) in differentiating between PD and non-PD cases, as evidenced in our study involving 214 PD and 208 non-PD skin samples within a 24-h timeframe. QSAA is a potential sensitive tool for exploring PD pathogenesis and for clinical diagnosis.

### Supplementary Information

**Additional file 1: Fig. S1** QSAA of mPFFs. **Fig. S2** Optimal conditions for in situ amplification of αSyn aggregates in brain tissue. **Fig. S3** Confocal analyses of QSAA amplified products. **Fig. S4** In situ amplification of αSyn aggregates in autopsy brain tissue. **Fig. S5** Comparison of VMAT2 distribution in healthy controls and Hoehn-Yahr stage 2-4 patients. **Table S1**. The demographic and clinical features of PD and non-PD controls. **Table S2.** The concordance between the SAA and QSAA assays in detecting αSyn seeding activity in PD brain homogenates.

## Data Availability

This paper does not report the original code. Any additional information required to reanalyze the data reported in this report is available from the lead contact upon request. All materials used in this study will be made available subject to a materials transfer agreement.

## Abbreviations

αSyn: Alpha-synuclein
AS: Ammonium sulfate
HM: Human monomer
hPFFs: Human pre-formed fibrils
LB: Lewy Body
MM: Mouse monomer
mPFFs: Mouse pre-formed fibrils
MSA: Multiple system atrophy
PD: Parkinson’s disease
PEG: Polyethylene glycol
PMCA: Protein misfolding cyclic amplification
QSAA: Quiescent seed amplification assay
RT-QuIC: Real-time quaking-induced conversion
SAA: Seed amplification assay
ThT: Thioflavin T
