# Supplementary material for: Ultrasensitive detection of aggregated α-synuclein using quiescent seed amplification assay for the diagnosis of Parkinson’s disease
Source: Transl Neurodegener. 2024 Jul 24;13:35. doi: 10.1186/s40035-024-00426-9 (PMC11267792; doi:10.1186/s40035-024-00426-9)
Supplement: Supplementary file 1 — Additional file 1: Fig. S1 QSAA of mPFFs. Fig. S2 Optimal conditions for in situ amplification of αSyn aggregates in brain tissue. Fig. S3 Confocal analyses of QSAA amplified products. Fig. S4 In situ amplification of αSyn aggregates in autopsy brain tissue. Fig. S5 Comparison of VMAT2 distribution in healthy controls and Hoehn-Yahr stage 2-4 patients. Table S1. The demographic and clinical features of PD and non-PD controls. Table S2. The concordance between the SAA and QSAA assays in detecting αSyn seeding activity in PD brain homogenates. [file 40035_2024_426_MOESM1_ESM.docx]

**Additional file 1.**


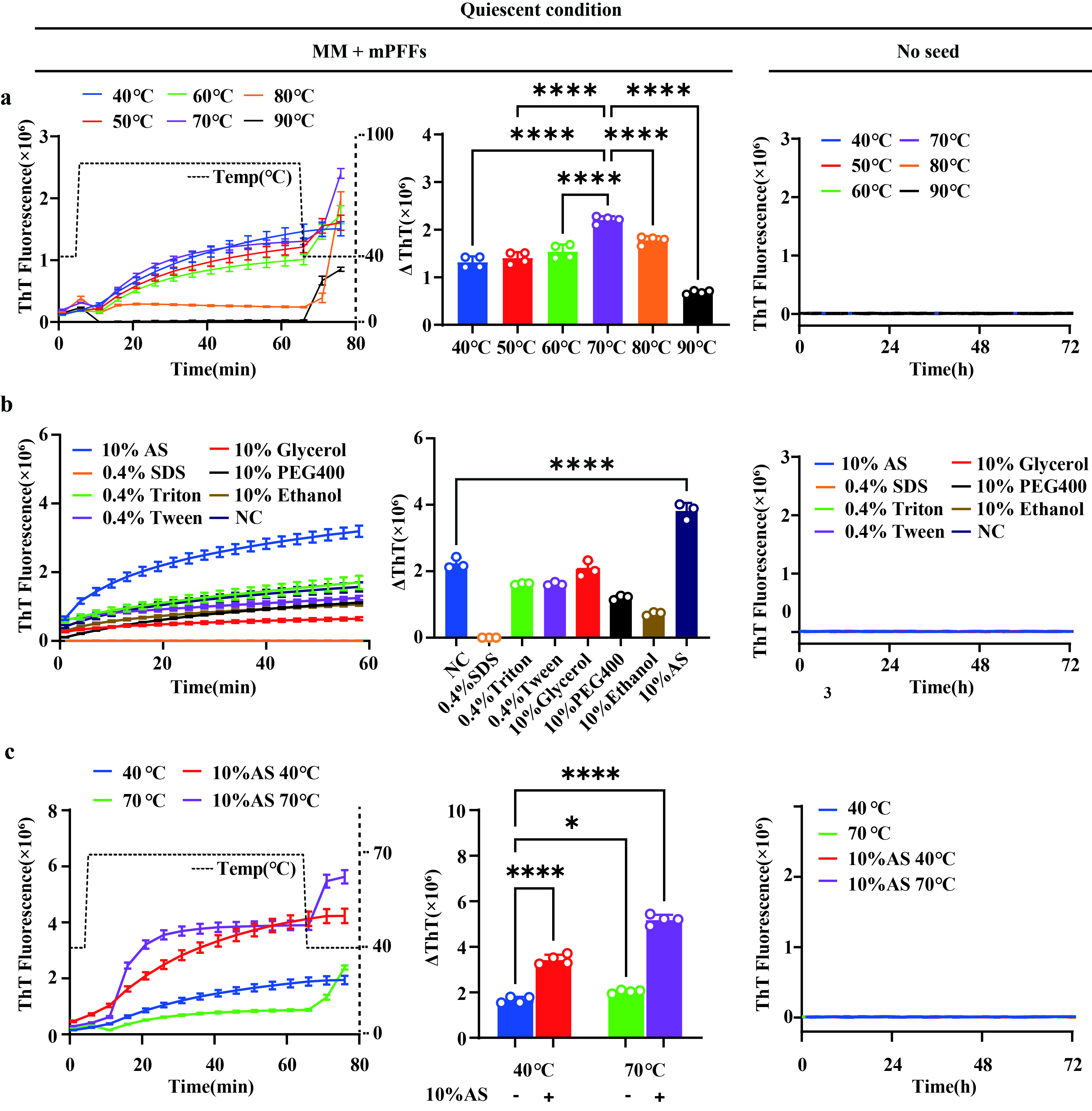


**Fig. S1** QSAA of mPFFs. The kinetics of fibril elongation were measured over time, while the bar chart corresponding to the curve shows the change of ThT values (ΔThT) after quiescent incubation. **a** The speed of the homologous reaction was increased over elevated temperature and then decreased when reaching the optimum temperature. The optimum reaction temperature for MM + mPFFs was 70°C. In control experiments conducted in the absence of PFFs (*n =* 4), neither of the incubation conditions demonstrated a substantial impact on the signal response. A temperature regression was conducted following amplification to correct for the impact of varying temperature settings on ThT fluorescence. **b** The effect of exogenous components (10% AS, 10% ethanol, 10% glycerol, 10% PEG-400, 0.4% SDS, 0.4% Triton X-100, or 0.4% Tween-20) on αSyn fibril elongation was detected by ThT fluorescence in quiescent conditions. In control experiments conducted in the absence of PFFs (*n =* 4), neither of the incubation conditions exhibited a significant effect on the signal response. **c** The elongation rates of MM was monitored by ThT fluorescence at 40°C or 70°C in the presence of 10% AS. In control experiments conducted in the absence of PFFs (*n =* 4), all incubation conditions showed no significant influence on the signal response. 0.01 mg/ml mPFFs was added to all of the above reaction conditions with seeds. A temperature regression was conducted following amplification to correct for the impact of varying temperature settings on ThT fluorescence. All data are presented as mean ± S.D. **P* < 0.05, ***P* < 0.01, ****P* < 0.001, *****P* < 0.0001 by one-way analysis of variance (ANOVA) followed by Tukey’s multiple comparison test.


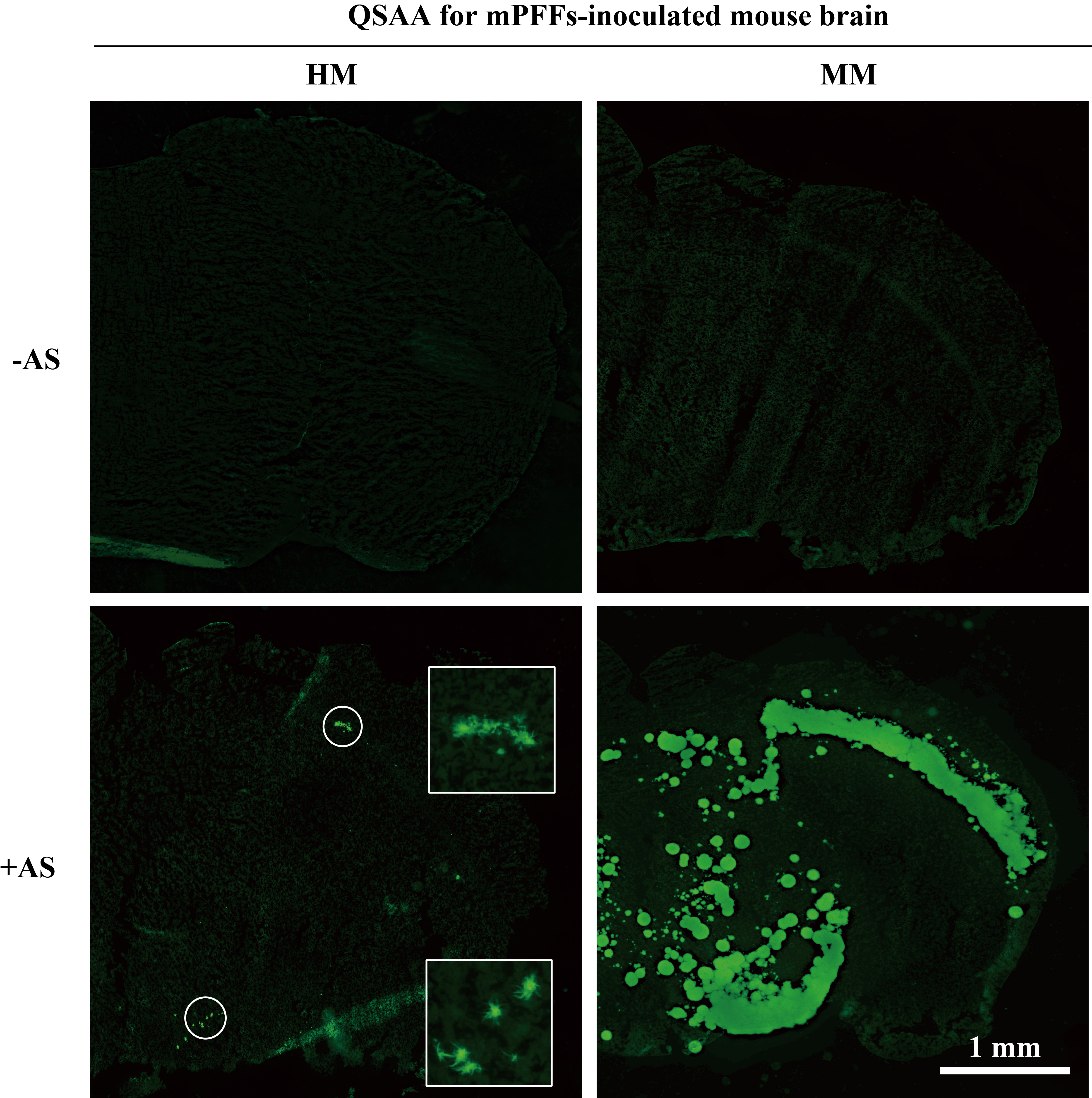


**Fig. S2** Optimal conditions for *in situ* amplification of αSyn aggregates in brain tissue. **a** Mouse brains were cut into 14 μm sections and incubated at 70°C for 12 h using QSAA (*n =* 6 per group). Fluorescence images were obtained after QSAA for 12 h in the presence of HM/MM with or without 10% AS. Strong circular fluorescent signals were visible only in the MM with 10% AS group, while the HM with 10% w/v AS group exhibited fewer filamentous fluorescent signals observed by fluorescence microscope. No positive signal was detected in the HM/MM without AS group. Scale bar = 1000 μm, for inserted box (40× magnification).


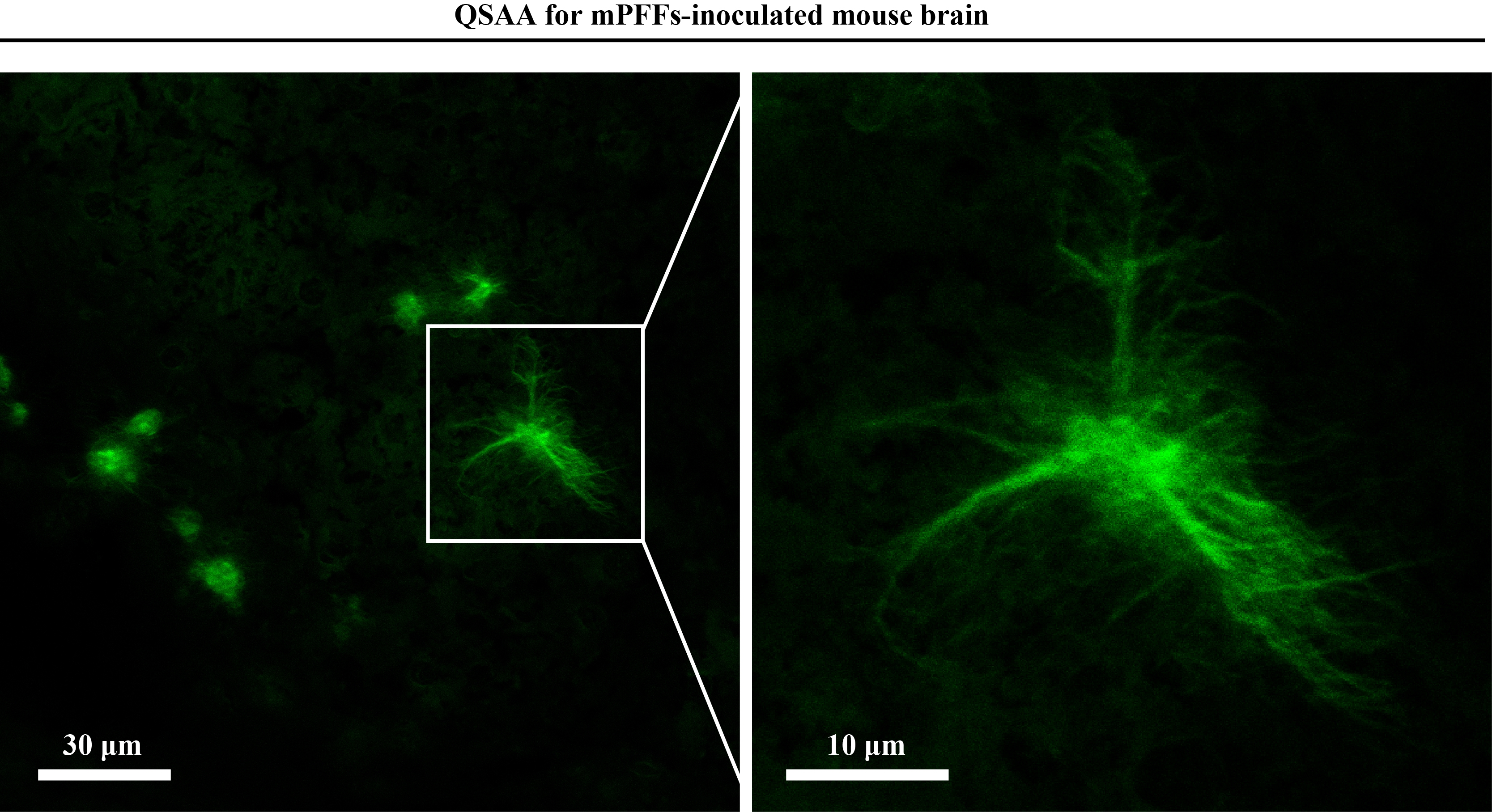


**Fig. S3** Confocal analyses of QSAA amplified products. Representative images of amplified QSAA products acquired from mPFFs-inoculated mouse brains after 12 hours of QSAA. Zoomed-in captions were captured using a 63 × lens.


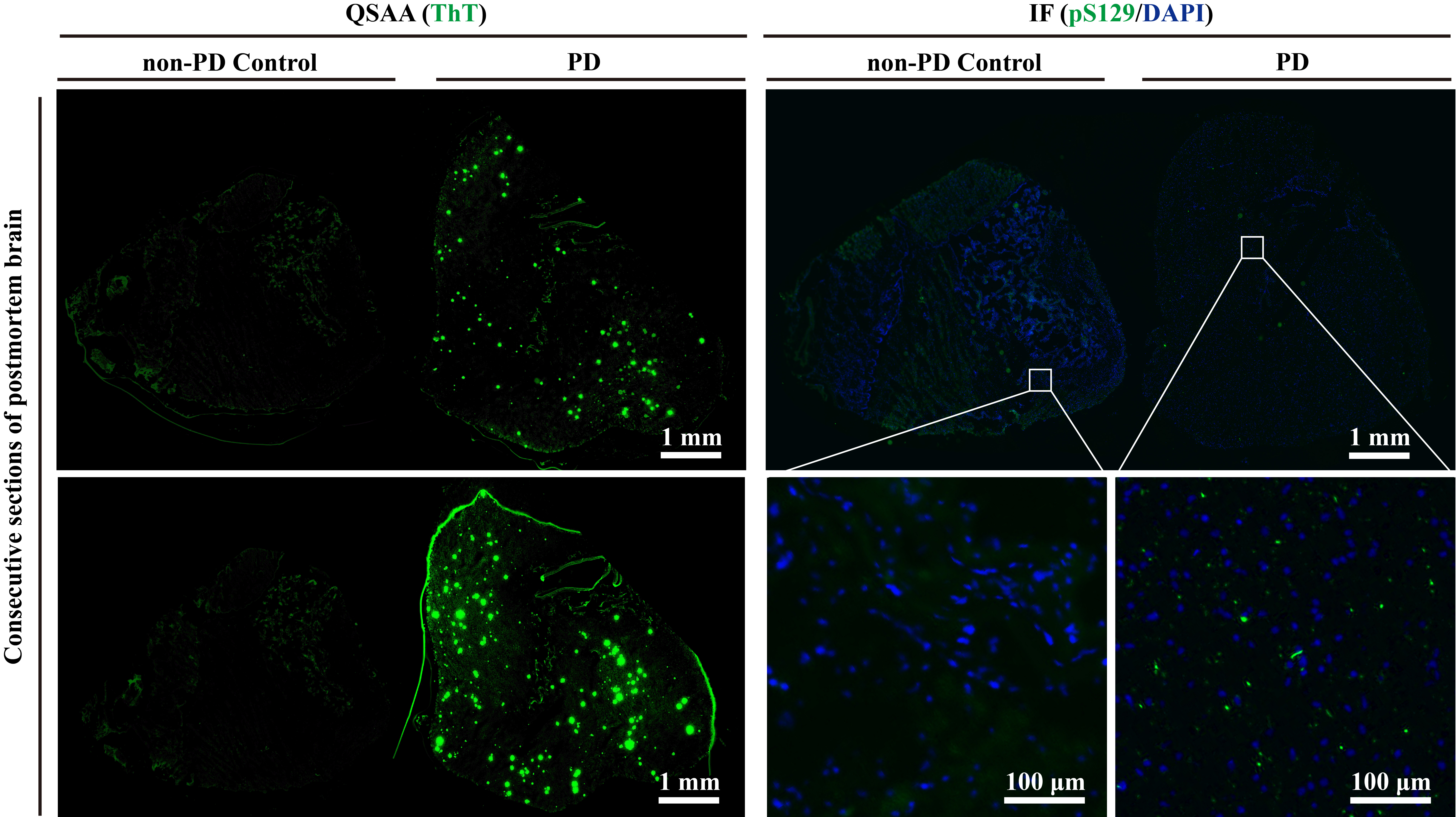


**Fig. S4** *In situ* amplification of αSyn aggregates in autopsy brain tissue. Representative images of pathological αSyn detected using both QSAA and IF (pS129) in PD and control brains. The top and bottom images in the QSAA panel represent two consecutive slices from a single subject. The zoomed captions in IF were obtained with a 63× lens.


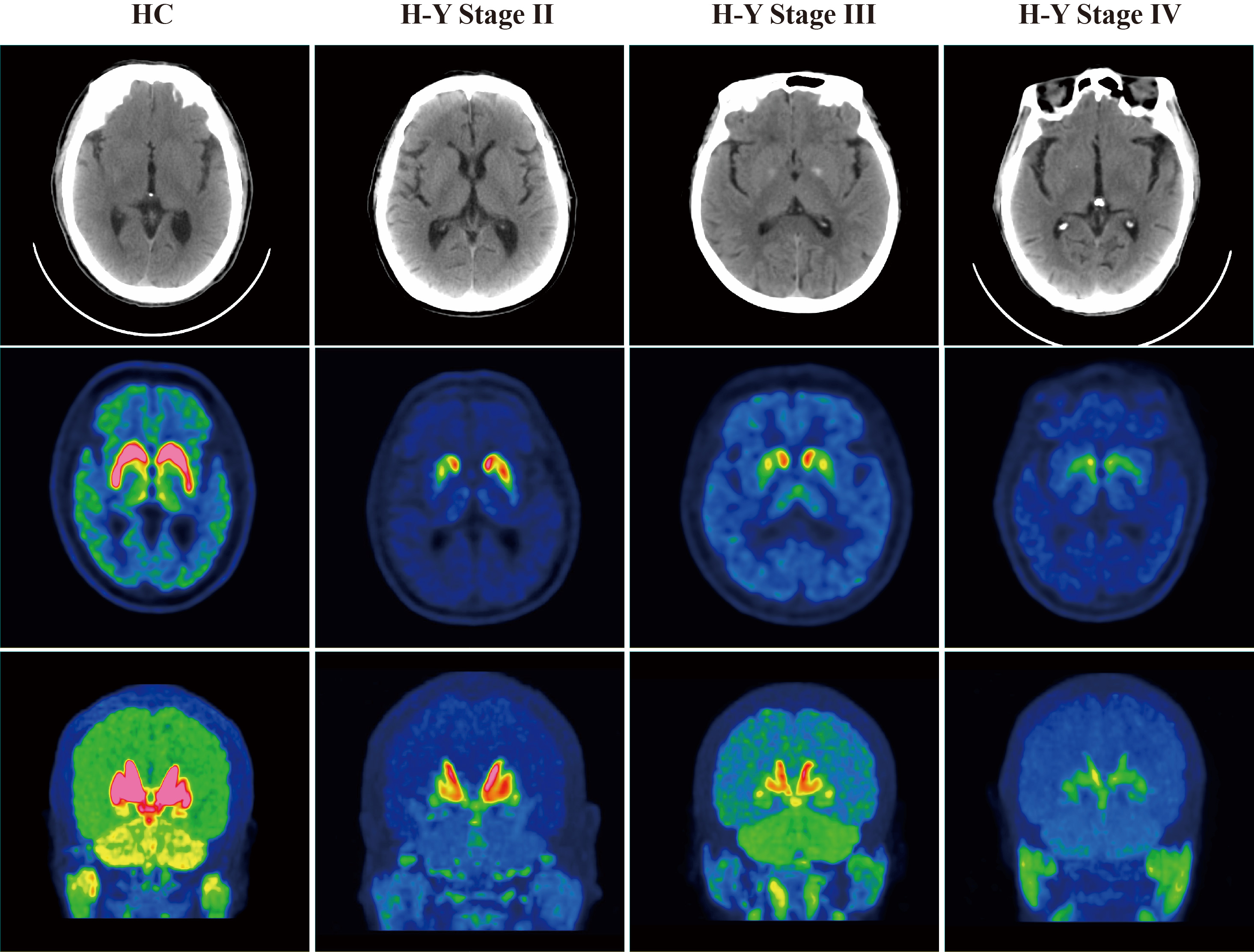


**Fig. S5** Comparison of VMAT2 distribution in healthy controls and Hoehn-Yahr stage 2-4 patients. Representative PET-CT image of VMAT2 distribution in healthy controls (*n =* 2) and patients (*n =*18) who scored within stages 2 to 4 on the Hoehn-Yahr scale (*n =* 18).

**Table S1. The demographic and clinical features of PD and non-PD controls.**

| **Case** | **Sex** | **Age** | **Disease duration** | **H-Y Stage** | **Clinical**  **diagnosis** |
| --- | --- | --- | --- | --- | --- |
|  |  | **[years]** | **[years]** | **[years]** |  |
| #1 | M | 63 | 7 | 3.5 | PD |
| #2 | F | 60 | 7 | 3 | PD |
| #3 | F | 59 | 8 | 3.5 | PD |
| #4 | M | 68 | 9 | 3.5 | PD |
| #5 | M | 69 | 11 | 4 | PD |
| #6 | M | 68 | 6 | 2 | PD |
| #7 | M | 69 | 7 | 3 | PD |
| #8 | M | 67 | 12 | 4 | PD |
| #9 | F | 73 | 8 | 3 | PD |
| #10 | M | 64 | 5 | 3 | PD |
| #11 | M | 62 | 5 | 2.5 | PD |
| #12 | F | 77 | 14 | 4.5 | PD |
| #13 | M | 64 | 5 | 3 | PD |
| #14 | F | 59 | 4 | 2.5 | PD |
| #15 | F | 43 | NA | NA | Epilepsy |
| #16 | M | 39 | NA | NA | Epilepsy |
| #17 | F | 40 | NA | NA | Epilepsy |
| #18 | F | 53 | NA | NA | Epilepsy |
| #19 | M | 36 | NA | NA | Epilepsy |
| #20 | M | 34 | NA | NA | Epilepsy |

**Table S2.** **The concordance between the SAA and QSAA assays in detecting αSyn seeding activity in PD brain homogenates.**

|  | **SAA** | **QSAA** | **Consistency** |
| --- | --- | --- | --- |
| **Positive** | 14 | 14 | 100% |
| **Negative** | 6 | 6 | 100% |
